# Down syndrome patients with normal hearts: are they really normal?

**DOI:** 10.1097/MD.0000000000032886

**Published:** 2023-02-10

**Authors:** Eman G Abdelrahman, Naglaa M Kamal, Sultan Alharthi, Muflih Albalawi, Effat Assar

**Affiliations:** a Pediatric department, Faculty of Medicine, Benha University, Benha, Egypt; b Pediatric department, Faculty of Medicine, Cairo University, Cairo, Egypt; c Pediatric department, Alhada Armed Forces Hospital, Taif, KSA; d Cardiology service department, King Salman Armed Forces Hospital, Tabuk, KSA.

**Keywords:** anatomical cardiac lesions, cardiac dysfunction, children, Down syndrome, heart, tissue Doppler

## Abstract

Even though congenital heart disease is a common finding in down syndrome (DS) patients, some of them have anatomically normal hearts. However, the term “normal” might not be suitable, as these patients usually suffer from functional cardiac dysfunction. Several research highlighted that despite the absence of anatomical heart defects, subtle cardiac function derangements are present in DS patients. We aim to assess cardiac functions by Two-dimensional echocardiography and tissue Doppler imaging (TDI) in pediatric DS patients who have anatomically normal hearts. One hundred seventy-two patients with karyotyping confirmed DS with anatomically normal hearts and 165 healthy normal control children were enrolled in the current study. Their cardiac functions were assessed using both 2-dimensional echocardiography and TDI. Both patients and controls had structurally and anatomically normal hearts. In DS patients, the right side of the heart showed a significant reduction in both systolic and diastolic functions. Systolic dysfunction was evident by significantly decreased levels of Tricuspid annular plane systolic excursion and systolic wave by TDI. Diastolic dysfunction of the right ventricle was evident by prolonged deceleration time by conventional echocardiography and a significant decrease in annular tissue doppler velocity during early diastole/late diastole ratio by TDI. The E/De ratio was significantly increased. Even with anatomically normal hearts, DS patients should undergo cardiac function assessment by echocardiography & TDI. TDI is superior to conventional echocardiography in detecting subtle cardiac dysfunction especially left ventricular diastolic dysfunction in DS patients. TDI showed a significant decrease in the early/atrial ratio of mitral valve annulus and prolongation of left ventricle isometric relaxation time in DS children. Also, the left ventricle E/De ratio was prolonged denoting elevated filling pressures and diastolic dysfunction. This indicates that the TDI has higher sensitivity to detect diastolic dysfunction than conventional Echocardiography. Biventricular TDI-derived myocardial performance index was found to be significantly increased in DS children.

## 1. Introduction

Down syndrome (DS) is well known for its high association with congenital heart disease with 40% to 60% of patients suffering from it.^[1,2]^ Some studies highlighted that even with anatomically normal hearts, functional cardiac dysfunction is always there in DS patients especially myocardial fibrillar structure abnormalities with subsequent diastolic ventricular dysfunction.^[1,3–5]^ Patients with DS frequently develop pulmonary hypertension in absence of anatomical defects due to abnormal growth of their pulmonary vasculature with subsequent derangement of cardiovascular capacity.^[6–10]^ Decreased exercise capacity was frequently noticed in adult DS patients with anatomically normal hearts with no clear understanding of the underlying pathogenesis.^[5]^

Studies with tissue Doppler Imaging (TDI) & speckle-tracking echocardiography allow for the early detection of subtle cardiac functional abnormalities.^[11]^

We aim to assess cardiac functions in pediatric DS patients, who have anatomically normal hearts. Examining cardiac function in this vulnerable population is critical to identify risk for decreased exercise capacity and increased risk for morbidity.

## 2. Patients and methods

### 2.1. Patients

We retrospectively reviewed medical records of the pediatrics, pediatric cardiology, and pediatric genetic outpatients’ clinics of Benha University Specialized Children Hospital, Benha, over the period between December 2018 and January 2022, for all DS patients who have an anatomically normal heart. Patients with DS and anatomically normal hearts were then prospectively evaluated thoroughly (refer to the methods section). For the control group, we recruited a comparable number of normally matched age and sex children.

The study was approved by the local institutional review board of Benha University. Written informed consents were obtained from the guardians of all patients and controls for the contribution to the study and for the of the study data.

### 2.2. Inclusion criteria

1. Pediatric and adolescents DS patients.
2. Age 6 months- 18 years.
3. DS genetically proven by karyotyping.
4. Normal chest X ray (CXR) & electrocardiogram (ECG).
5. Anatomically normal heart by echocardiography (No congenital or acquired anatomical cardiac defects).
6. Negative history of chronic diseases.

### 2.3. Exclusion criteria

1. Associated congenital or acquired heart disease.
2. Associated pulmonary hypertension of any cause.
3. Any chronic pulmonary disease or upper airway obstruction.
4. Uncontrolled bronchial asthma.
5. Peripheral or central hypoventilation.
6. Thoracic skeletal deformities.
7. Obstructive sleep apnea excluded by the sleep studie.
8. Tonsillo-adenoidal hypertrophy.
9. Hypothyroidism.

## 3. Methods

### 3.1. All patients and controls were subjected to the following

1. Full history taking: Age, sex, maternal age, cardiac symptoms, and other body symptomatology review.
2. Thorough clinical examination: Anthropometric measurements (weight, height & body mass index [BMI]), vital signs (heart rate [HR], respiratory rate [RR], systolic & diastolic blood pressures [BP]), and ear, nose, throat, respiratory, cardiovascular, and other body systems examination.
3. Radiological workup by CXR.
4. ECG: for detection of any cardiac arrhythmia.
5. Conventional echocardiography:

Detailed conventional echocardiography was performed at the Pediatric Cardiology Unit, Benha University Hospitals. It was done using Hewlett Packard/Phillips SONOS 5500 systems–Palo Alto, California – equipped with 2.5 to 5 megahertz transducers.

It included measurements of the following:

1. Left ventricular dimensions at end-systole and end-diastole, interventricular septum, ejection fraction (EF) estimated by cube method, and fraction shortening (FS) evaluated by M-mode echocardiography in the parasternal short axis. All measurements were compared to standard tables for ages and body surface area.^[12]^ An EF of < 55% was considered abnormal and FS of < 29% was considered abnormal.^[11]^
2. Mitral & tricuspid Doppler signals evaluated in the apical 4-chamber view: early diastolic peak velocity (E, cm/s), mitral deceleration time of early filling (DCC) in milliseconds, late diastolic peak velocity (A, cm/s), and early/late diastolic peak velocity ratio (E/A).
3. Assessment of pulmonary artery (PA) systolic pressure via tricuspid regurgitation jet using the Bernoulli equation.
4. Assessment of mitral annular plane systolic excursion using the M-mode in apical 4-chamber view & tricuspid annular plane systolic excursion (TAPSE) using the M-mode in apical 4-chamber view, placing the examination beam on the lateral mitral & tricuspid annuluses.

1. Tissue-Doppler-Imaging:

TDI was obtained from an apical 4-chamber view to obtain longitudinal annular velocities at the lateral mitral wall, septum, and lateral tricuspid wall adjacent to the atrioventricular valve hinge points. annular tissue doppler velocity during systole (Sa), early diastolic (De), and late diastolic (Da) tissue Doppler velocities, isovolumetric contraction, and relaxation times will be measured at the lateral mitral, septal, and lateral tricuspid walls. Trans mitral and trans tricuspid E/Ea ratios, ejection time for anterior wall, posterior wall, and septum, and myocardial performance index for them were calculated for each patient.

### 3.2. Statistical analysis

SPSS version 22 was used to tabulate and analyze the gathered data (SPSS Inc, Chicago, ILL Company). Percentages and numbers were calculated for categorical data. The Chi-square test (X2), or Fisher exact test was used to analyze categorical variables. Quantitative data were tested for normality using the Kolmogorov-Smirnov test assuming normality at *P* > .05.

Variables such as means, standard deviations, and ranges were used for quantitative data. Mann–Whitney U test was used for nonparametric variables and the student *t* test was used to evaluate regularly distributed variables between 2 independent groups. The accepted level of significance was 0.05 (*P* < .05 was considered significant).

## 4. Results

Over the study period, 172 genetically proven pediatric DS patients fulfilling the study inclusion criteria were enrolled in the current study. All patients had normal CXR, ECG, and absent anatomical cardiac lesions by echocardiography as prerequisites for enrollment. One hundred sixty-five healthy children who were age and sex-matched served as the control group.

At the time of enrollment in the study, the demographic data of the patients and controls were comparable with no statistical differences. The maternal age of patients was statistically significantly higher than that of controls (*P* < .001) (Table 1).

**Table 1:** Demographic and clinical characteristics of patients and controls.

Parameter		Patients (n = 172)	Controls (n = 165)	*P* value
**Age (yr**) Median (range)		4.8 (0.62–16.63)	6.6 (0.67–17.34)	.203
**Gender No. (%**)	Males	100 (58.0)	92 (56.0)	.878
	Females	72 (42.0)	73 (44.0)	.981
**Maternal age (yr**) Mean ± SD		35 ± 5	25 ± 1	<.001*
**Weight (kg**) Median (range)		12.1 (4.9–54.2)	22.8 (6.8–34.7)	.313
**Height (cm**) Median (range)		79.2 (61.7–153.6)	121.3 (69.4–130.9)	.030*
**Body mass index (Kg/m^2^**) Median (range)		19.3 (12.8–22.9)	15.5 (14.1–20.3)	.005*
**Heart rate/min**		84.3 ± 7.8	80.8 ± 7.2	<.001*
**Respiratory rate/min**		21.7 ± 2.4	20.3 ± 2.4	<.001*
**Systolic Blood pressure (mm Hg**)		100.7 ± 8.4	102.8 ± 8.8	.026*
**Diastolic Blood pressure (mm Hg**)		59.5 ± 4.9	60.2 ± 5.6	.222

* Significant.

DS patients had significantly shorter height than controls, while their BMI was significantly higher than that of the control group (Table 1). DS patients have statistically higher HR & RR than controls. Their systolic BP was significantly lower while their diastolic BP was insignificantly lower than the controls (Table 1).

Comparison of the echocardiographic assessment of the left ventricle (LV) in patients and controls revealed, significantly lower left ventricular end-diastolic dimensions & left ventricular end-systolic dimensions dimensions, while their EF, FS, and E & A velocities were significantly higher than that of controls (Table 2).

**Table 2:** Comparison of Echocardiographic Findings between Patients and Controls.

Parameter	Patients (n = 172)	Controls (n = 165)	*P* value
**Left ventricle parameters**			
**LVED (z score**) Median (range)	−1.5 (−2.5 to 0.5)	0.7 (0–1.5)	<.001*
**LVES (cm)** (Mean ± SD)	1.71 ± 0.74	2.15 ± 0.59	.012*
**EF (%**) Mean ± SD	73.4 ± 7.2	71 ± 1.7	<.001*
**FS (%**) Mean ± SD	41.1 ± 6.6	39.2 ± 1.2	<.001*
**E Velocity (Cm/s**) Mean ± SD	107.6 ± 20.1	92.4 ± 13.4	<.001*
**A Velocity (Cm/s**) Mean ± SD	69.1 ± 16.2	53.9 ± 6.2	<.001*
**E/A ratio** Mean ± SD	1.7 ± 0.6	1.7 ± 0.1	1.000
**DCC time (ms**) Mean ± SD	138 ± 21	136 ± 40	.564
**Right ventricle parameters**			
**TAPSE (mm**) **Mean ± SD**	14.6 ± 4	17.1 ± 2.7	< .001*
**E velocity (Cm/s**) Mean ± SD	83.5 ± 15.6	71.5 ± 14.4	<.001*
**A Velocity (Cm/s**) Mean ± SD	60.2 ± 11.1	47.3 ± 8	<.001*
**E/A ratio** Mean ± SD	1.4 ± 0.3	1.5 ± 0.2	.870
**DCC Time(ms**) Mean ± SD	160 ± 35	142 ± 2	<.001*
**Pulmonary artery parameters**			
**TR (mm Hg**) Mean ± SD	28 ± 6	17 ± 5	<.001*
**EPASP (mm Hg**) Mean ± SD	38 ± 6	27 ± 5	<.001*

A = late diastolic inflow peak velocity, DCC time: deceleration time, E = early diastolic inflow peak velocity, EF = Ejection fraction, EPASP = estimates pulmonary artery systolic pressure, FS = fractional shortening, LVED = left ventricular end-diastolic dimensions, LVES = left ventricular end-systolic dimensions, TAPSE = tricuspid annular plane systolic excursion, TR = tricuspid regurgitation.

* Significant.

On the other hand, a comparison of the echocardiographic assessments of the right ventricle (RV) in patients and controls revealed, significantly lower TAPSE while the E & A velocities, the right ventricle DCC time, tricuspid regurgitation, and EPASP were significantly higher in patients than controls (Table 2).

No intergroup differences were detected regarding left ventricular DCC time and E/A ratio for both LV & RV (Table 2).

TDI evaluation of the LV showed that; LV isovolumetric contraction and relaxation times, E/De ratio, and MPI were significantly higher in patients. The mitral annulus Sa wave, De wave, De/Da ratio, and ejection time were significantly lower in patients compared to controls (Table 3).

**Table 3:** Comparison of the left ventricle tissue Doppler findings between patients and controls.

Parameter	Down syndrome	Control group	*P* value
Mitral annulus Sa wave (cm/s) (Mean ± SD)	7.11 ± 1.54	7.52 ± 1.49	.014*
Mitral annulus De wave (cm/s) (Mean ± SD)	11.89 ± 2.59	12.82 ± 2.61	.001*
Mitral annulus Da wave (cm/s) (Mean ± SD)	7.24 ± 1.8	7.06 ± 0.92	.252
Mitral valve De/Da wave	1.64 ± 1.43	1.82 ± 2.1	<.001*
Isovolumetric contraction time (ms) (Mean ± SD)	42 ± 8	37 ± 7.51	<.001*
Isovolumetric relaxation time (ms) (Mean ± SD)	49 ± 5	42 ± 7	<.001*
Ejection time (ms) (Mean ± SD)	242 ± 30	261 ± 14.52	<.001*
Left ventricle E/De (Mean ± SD)	9.05 ± 1.82	7.22 ± 5.15	<.001*
MPI of the left ventricle (Mean ± SD)	0.38 ± 0.12	0.31 ± 0.05	<.001*

Da = Late diastolic annular tissue Doppler velocity, De = early diastolic annular tissue Doppler velocity, E/De = ratio between early diastolic inflow peak velocity and early diastolic annular tissue Doppler velocity, MPI = myocardial performance index, ms = milliseconds, Sa = systolic annular tissue Doppler velocity.

* Statistically significant.

TDI evaluation of the RV showed that; the tricuspid annulus Sa & De waves, De/Da ratio, and RV ejection time are significantly lower in patients as compared to controls. On the other hand, patients had significantly higher tricuspid annulus Da wave, RV isovolumetric contraction & relaxation times, E/De ratio, and MPI (Table 4).

**Table 4:** Comparison of the right ventricle tissue Doppler findings between patients and controls.

Parameter	Down syndrome	Control group	*P* value
Tricuspid annulus Sa wave (mm/Hg) (Mean ± SD)	10.22 ± 3.53	10.79 ± 1.15	.049*
Tricuspid annulus De wave (mm/Hg) (Mean ± SD)	13.12 ± 0.41	15.05 ± 3.84	<.001*
Tricuspid annulus Da wave (mm/Hg) (Mean ± SD)	10.22 ± 4.99	8.29 ± 0.91	<.001*
Tricuspid valve De/Da wave	1.28 ± 0.11	1.81 ± 0.62	<.001*
Isovolumetric contraction time (ms) (Mean ± SD)	45 ± 9	40 ± 3	<.001*
Isovolumetric relaxation time (ms) (Mean ± SD)	46 ± **7**	42 ± 10	<.001*
Ejection time (ms)	257 ± 33	281 ± 28	<.001*
Right ventricle E/De	6.37 ± 1.41	4.75 ± 1.32	<.001*
MPI of the right ventricle	0.35 ± 0.11	0.29 ± 0.05	<.001*

Da = Late diastolic annular tissue Doppler velocity, De = early diastolic annular tissue Doppler velocity, E/De = ratio between early diastolic inflow peak velocity and early diastolic annular tissue Doppler velocity, MPI = myocardial performance index, ms = milliseconds, Sa = systolic annular tissue Doppler velocity.

* Statistically significant.

No intergroup difference was detected regarding the mitral annulus Da wave (Table 3).

## 5. Discussion

DS is one of the most common chromosomal abnormalities in humans characterized by a wide phenotypic variation of multiple cardiac as well as systemic complications.^[13]^ Owing to the fact that this causes a high burden on the community and health care system and the high prevalence of DS patients worldwide, there is an urgent necessity for a proper integrated follow-up and management plan for those patients.

Cardiac assessment is core care in all DS patients due to the high association with congenital heart disease (40%–60%)^[1]^ which in turn affects their quality of life. It was found that even in absence of congenital cardiac defects, DS patients have diminished cardiac capacity.^[5,14]^ This might be attributed to their baseline-associated hypotonia and to subtle cardiac functions abnormalities, myocardial fibrillar structure, and autonomic nervous system dysfunctions even in absence of anatomical congenital cardiac defects.^[1,14]^ Pastore et al,^[15]^ 2000 showed that children with DS without congenital heart disease have a reduced tolerance for exercise, higher heart rates, and higher arterial blood pressure. However, no symptoms (syncope, chest pain, or dyspnea) or arrhythmias occurred during exercise.

We carried out this study to assess cardiac dysfunction in DS patients with anatomically normal hearts. All patients underwent both 2-dimensional- echocardiography and TDI. One hundred seventy-two DS patients fulfilling the study inclusion criteria were enrolled in the study. Their median age was 4.8 years (range: 0.62–16.63) which was younger than the age reported in the previous studies.^[1,16,17]^ This might be explained by a better understanding of the physicians & better education of the parents that regular follow-up of DS patients is mandatory despite the absence of cardiac abnormalities. Previously, it was assumed that in the absence of anatomical cardiac defects, no cardiological follow-up was needed which contributed to the delayed presentation of functional cardiac abnormalities at older ages in previous studies. Hospitals should have integrated DS clinics providing multidisciplinary care plans for the different health needs of DS patients.

Fifty-eight percent of our patients were males with a male-female ratio of 1.4:1, which was statistically insignificant. A similar finding of 1.3:1 & 1.9:1 was reported before by Al-Biltagi et al^[16]^ & Balli et al^[1]^ respectively, while Youssef & Raouf^[17]^ had a reverse ratio (0.8: 1). No clear significance/explanation of those finding was found in the literature.

The significantly higher maternal age in the DS group is a well-known association with DS detected in our report and in all previous literature.^[18,19]^ The significantly shorter height in the DS group was also expected as an invariable feature of DS.

We agreed with Al-Biltagi et al^[16]^ in having significantly higher BMI in the DS group. Balli et al^[1]^ detected insignificant higher BMI in their DS patients. The higher BMI may be related to decreased physical activity of DS patients.

Assessment of the vital signs revealed, significantly higher HR & RR & lower systolic BP in the DS group, while they had insignificantly lower diastolic BP. Those findings were also reported in previous studies.^[1,16,17]^ Santoro et al,^[20]^ 2020 found that there is a 12-percentile point reduction in baseline BP in pediatric DS patients with normal hearts compared with age- and height-matched controls as reported in the standard national heart, lung, and blood institute/national health and nutrition examination survey and AAP cohorts. This might be explained by the associated autonomic cardiac dysregulation in DS patients due to the associated brain stem central autonomic dysfunction in DS patients.^[21]^ This has also been observed in the form of blunted heart rate and blood pressure responses to tilt-table testing.^[22]^ The lower blood pressure in those patients might be related to the trisomy of the type-1-angiotensin II receptor gene, resulting in its repression.^[23]^

Regarding conventional Echocardiography, the LV EF & FS were significantly higher in DS children. These results were similar to previous reports.^[1,16]^ This hyperkinesia is mostly attributed to the reduced afterload in DS patients due to trisomy of the type-1-angiotensin II receptor gene.

However, the presence of intrinsic myocardial abnormalities in DS patients, even with structurally normal hearts was proven by subtle systolic dysfunction detected by TDI.

Sa wave on the mitral valve was significantly decreased in our DS cohort as compared to controls with values of 7.11 cm/s and 7.52 cm/s respectively with a *P* value of .01. Isovolumetric contraction time was significantly prolonged in children with DS with a *P* < .001.

Sa wave is less load dependent, whereas the isometric contraction time (ICT) is inversely correlated with myocardial contractility. Al- Biltagi et al^[16]^ demonstrated a lack of significant difference between systolic wave and ICT of the mitral valve annulus measured by TDI between DS children and the controls.

Our results showed an insignificant difference between the E/A ratio or DCC on the mitral valve by the conventional Echocardiography with *P* values 1.0 and 0.5 respectively. These results were conflicting with Balli et al^[1]^ who found a significant decrease in the E/A ratio on the mitral valve in DS children compared to controls.

However, TDI showed a significant decrease in the early/atrial ratio of mitral valve annulus and prolongation of LV isometric relaxation time in the DS children than in the controls (*P* < .001). Moreover, the LV E/De ratio was prolonged denoting elevated filling pressures and diastolic dysfunction. This may be due to the higher sensitivity of the TDI to detect diastolic dysfunction in comparison to conventional Echocardiography.

Diastolic dysfunction in DS is most probably due to impaired cardiac muscle relaxation. This was agreed with previous reports.^[1,17]^

Children with DS are at increased risk of developing PA hypertension even without structural heart lesions. In the current study, the estimated systolic PA pressure was significantly higher in children with DS. This can be explained by abnormal pulmonary vasculature, alveolar hypoventilation, and recurrent pulmonary infections.^[24]^ We excluded patients with chronic upper airway obstruction from our cohort.

The right side of the heart showed significant reduction in both systolic and diastolic functions by conventional Echocardiography and TDI on the tricuspid valve. Systolic dysfunction was evident by significantly decreased levels of TAPSE and Sa by TDI. ICT was also prolonged significantly with a *P* < .001.

Diastolic dysfunction of the RV was detected by prolonged DCC by conventional echocardiography and a significant decrease in De/Da ratio by TDI. The isometric relaxation time & RV E/De ratio was significantly increased. These results are concordant with Al Biltagi and coresearchers,^[16]^ who concluded that the reduction of the systolic and diastolic cardiac functions in children with DS may be explained by cardiac autonomic dysregulation, increased cardiac muscle fiber size, reduced cell number, and overexpression of calcineurin.

B-ventricular TDI-derived myocardial performance index was found to be significantly increased in DS children compared to controls, which confirms subclinical ventricular dysfunction in DS children with anatomically and clinically normal hearts. This was agreed with Abtahi and coauthors.^[25]^

In conclusion, even with anatomically normal hearts, DS patients should undergo cardiac functions assessment by both echocardiography & TDI. TDI is superior to conventional echocardiography in detecting subtle cardiac dysfunction especially left ventricular diastolic dysfunction in DS patients.

## Author contributions

**Conceptualization:** Eman G Abdelrahman.

**Data curation:** Eman G Abdelrahman, Effat Assar.

**Formal analysis:** Eman G Abdelrahman.

**Investigation:** Eman G Abdelrahman, Effat Assar.

**Methodology:** Effat Assar.

**Validation:** Eman G Abdelrahman.

**Writing – original draft:** Eman G Abdelrahman, Naglaa M Kamal, Sultan Alharthi, Muflih Albalawi, Effat Assar.

**Writing – review & editing:** Eman G Abdelrahman, Naglaa M Kamal, Sultan Alharthi, Muflih Albalawi.
